# A Stripline-Based Planar Wideband Feed for High-Gain Antennas with Partially Reflecting Superstructure

**DOI:** 10.3390/mi10050308

**Published:** 2019-05-07

**Authors:** Affan A Baba, Raheel M Hashmi, Mohsen Asadnia, Ladislau Matekovits, Karu P Esselle

**Affiliations:** 1 School of Engineering, Macquarie University, Sydney, New South Wales 2109, Australia; raheel.hashmi@mq.edu.au (R.M.H.); mohsen.asadnia@mq.edu.au (M.A.); karu.esselle@mq.edu.au (K.P.E.); 2 Department of Electronics and Telecommunication, Politecnico di Torino, 10129 Turin, Italy; ladislau.matekovits@polito.it

**Keywords:** high-gain, compact, wideband, resonant cavity, Fabry–Perot cavity, cavity resonator, EBG resonator

## Abstract

This paper presents a new planar feeding structure for wideband resonant-cavity antennas (RCAs). The feeding structure consists of two stacked dielectric slabs with an air-gap in between. A U-shaped slot, etched in the top metal-cladding over the upper dielectric slab, is fed by a planar stripline printed on the back side of the dielectric slab. The lower dielectric slab backed by a ground plane, is used to reduce back radiation. To validate the wideband performance of the new structure, in an RCA configuration, it was integrated with a wideband all-dielectric single-layer partially reflecting superstructure (PRS) with a transverse permittivity gradient (TPG). The single-layer RCA fed by the U-slot feeding structure demonstrated a peak directivity of 18.5 dBi with a 3 dB directivity bandwidth of 32%. An RCA prototype was fabricated and experimental results are presented.

## 1. Introduction

Common approaches to achieve high antenna directivity include the use of antenna arrays, which require a complex feeding network, or reflector antennas, which are bulky in nature. Resonant-Cavity Antennas (RCAs) have been investigated extensively as a convenient alternative in applications requiring directivity in the range of 15–25 dBi [1,2,3,4]. An RCA is formed by placing a partially reflecting superstructure (PRS) in front of a primary source antenna (such as a slot, or dipole), above a metallic ground plane or Artificial Magnetic Conductor (AMC) [5]. The directivity of this source antenna is significantly increased by multiple reflections between the PRS and the ground plane. The PRS can take various forms, depending on the design approach and fabrication methods used to construct the RCAs, including 3-D Electromagnetic Band Gap (EBG) structures [6]; 2-D metallo-dielectric frequency-selective surfaces [7]; or stacks of unprinted dielectric slabs [8]. Thus, RCAs are also referred to as EBG resonator antennas [9], Fabry–Perot cavity antennas [10], and 2D leaky-wave antennas [11].

**Figure 1:**
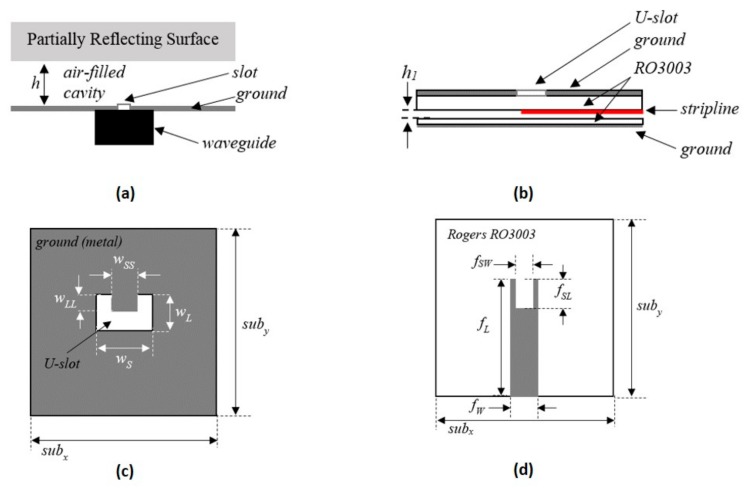
Structural schematic of the proposed planar feed, (**a**) configuration of wideband resonant-cavity antennas (RCAs) fed using conventional waveguides, (**b**) vertical cross section of the antenna, showing the stripline and the air-gap, (**c**) top view of the antenna, showing the U-slot cut in the ground plane, (**d**) horizontal cross section of the antenna, showing the stripline.

A number of wideband RCAs having all-dielectric PRSs have been proposed with peak gain and directivity greater than 15 dBi and 3 dB directivity bandwidths that are significantly greater than the initial RCAs (1–3%) [12,13]. However, with the increase in bandwidth, the input matching becomes increasingly challenging. This effect is more pronounced when the peak gain of the RCAs ranges between 15–20 dBi, which is required in most medium-range applications. Therefore, rectangular slots cut in a ground plane, fed by waveguide-to-SMA adaptors (shown in Figure 1a) are commonly used to feed wideband high-gain RCAs, particularly when the bandwidths exceed beyond 20% [14,15,16,17,18,19]. However, standard waveguide adaptors are quite expensive, and mechanically bulky. Although they present a reasonable feeding method for antenna testing and characterization, their use adds significant cost, weight, and volume in most practical RCAs.

In this paper, a planar, wideband feed antenna is proposed, which can be used for feeding wideband RCAs. It can easily be fabricated through the standard-printed-circuit board (PCB) manufacturing process, and can easily be interfaced with any type of wideband PRS, to form a stand-alone RCA. Initially, the planar wideband feed antenna itself was numerically designed and studied using the Computer Simulation Technology Microwave Studio (CST MWS) time-domain solver. Once the desired performance was obtained, it was interfaced with a wideband dielectric PRS developed in Reference [16] and an RCA was designed using the CST-MWS time-domain solver. The RCA fed by this planar feed demonstrated a peak gain of 18.5 dBi with a 3 dB gain-bandwidth of 32%. A prototype was fabricated and measured to validate the performance

## 2. Design of Planar Wideband Feed Antenna

Figure 1b–d show the schematic of the proposed planar, wideband feed antenna. It consists of two substrates in a stacked configuration, with an air-gap in between (Figure 1b). The permittivity of the substrates is *ε_r_* = 3.02 and the loss tangent is tan δ = 0.0019. The top side of the upper substrate is metal-cladded, with a U-slot feed etched in the center (see Figure 1c). A stripline is etched on the bottom side of the upper substrate with a U-shaped stub added at the coupling end (see Figure 1d). The lower substrate has no metal cladding on the top side, but is backed by fully-metallic cladding. The lower substrate is placed at a distance h_1_ away from the stripline, and circumvents back radiation, as shown in later sections. All the design parameters shown in Figure 1 are given in Table 1.

**Figure 2:**
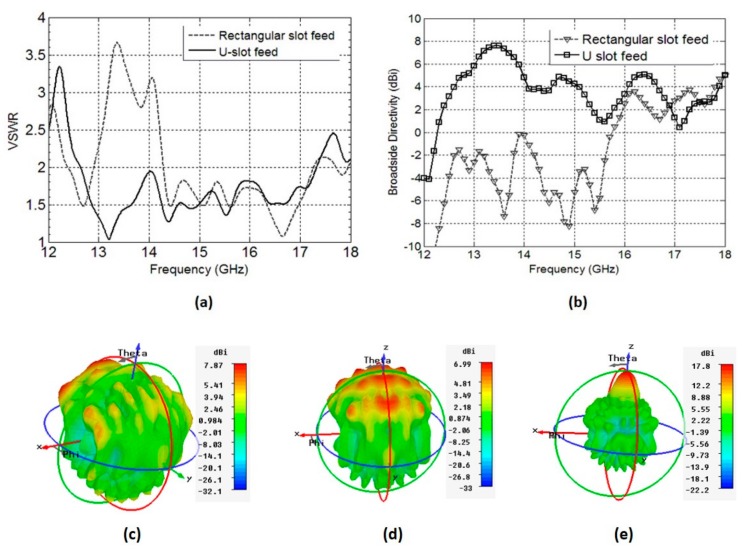
Comparison of rectangular slot with U-slot, when fed by the stripline, (**a**) voltage standing wave ratio (VSWR) of the rectangular slot and U-slot antenna, (**b**) broadside directivity of the rectangular slot and U-slot antenna, (**c**) radiation patterns of rectangular slot antenna and (**d**) radiation patterns of U-slot antenna (**e**) radiation patterns of the RCA with U-slot antenna at 15 GHz.

The RCA fed by a waveguide-to-SubMiniature version A (SMA) transition in [16] covers the entire Ku-band, with a peak gain of 20.7 dBi. Therefore, we selected this frequency band as a reference to replace the waveguide-to-SMA transition with a planar feed. Initially, we began with a simple rectangular slot fed by a stripline. The length and width of the rectangular slot were 13.125 mm and 9 mm, respectively. This configuration provided a VSWR 2:1 bandwidth extending from 14.2 GHz–17.3 GHz, providing matching in the upper Ku-band (see Figure 2a). Another important aspect to consider is that a rectangular slot fed by a waveguide-to-SMA adaptor, as done in Reference [16], provides a broadside-directed radiation pattern over the entire matched bandwidth. This characteristic is crucial to achieving high gain from an RCA. The rectangular slot fed by a stripline, however, does not radiate towards the broadside over the entire VSWR 2:1 bandwidth, as shown in Figure 2b,c. To improve the coupling towards the broadside, the rectangular slot was modified to form a U-slot, which directed the maximum radiation towards the broadside (see Figure 2b,d), and improved the VSWR 2:1 bandwidth to extend from 12.4 GHz to 18.2 GHz, which covers nearly the entire Ku-band. Extensive parametric studies were carried out to tune the performance of the U-slot and the stripline to achieve the optimal performance, but only the key results are presented and discussed here for brevity. The final values of the parameters that provide the best compromise between VSWR 2:1 bandwidth and broadside-directed radiation are shown in Table 1.

## 3. Resonant-Cavity Antennas (RCA) Design and Interfacing with Planar Feed

**Figure 3:**
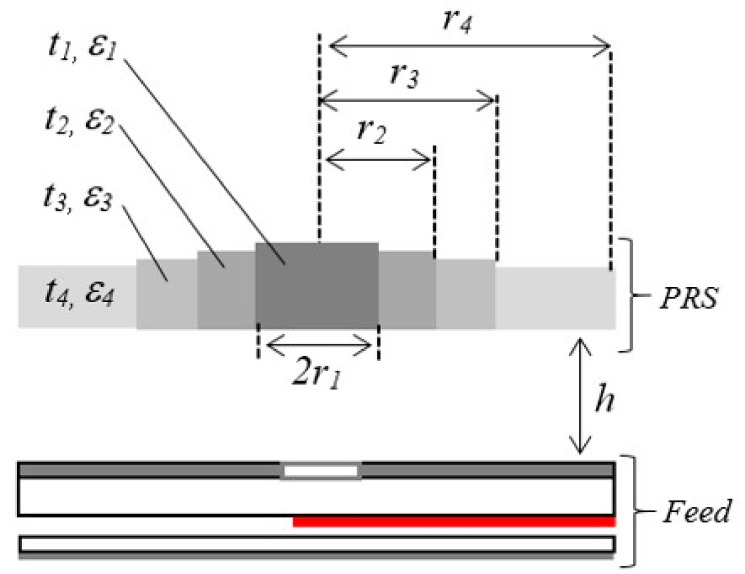
Schematic of RCA constructed by placing the wideband dielectric partially reflecting superstructure (PRS) at a height *h* above the proposed planar strip-line fed U-slot.

In this section, we describe the interfacing of the planar feed with a wideband dielectric PRS to design an RCA. The schematic of the wideband dielectric PRS is shown in Figure 3. The PRS has a circular shape and a diameter D = 78 mm (2.6λ_0_ at 10 GHz) and is placed at a distance h = 16 mm (~0.53λ_0_) from the top part of the feed antenna, as shown in Figure 3. In this configuration, the metallic cladding on the top side of the upper substrate of the planar feed antenna acts as a ground plane for the RCA. It is worth pointing out that the ground plane is essential to obtain wideband gain enhancement from this RCA, which was thoroughly studied and discussed in Reference [16]. The complete design parameters of the dielectric PRS are given in Table 2.

**Figure 4:**
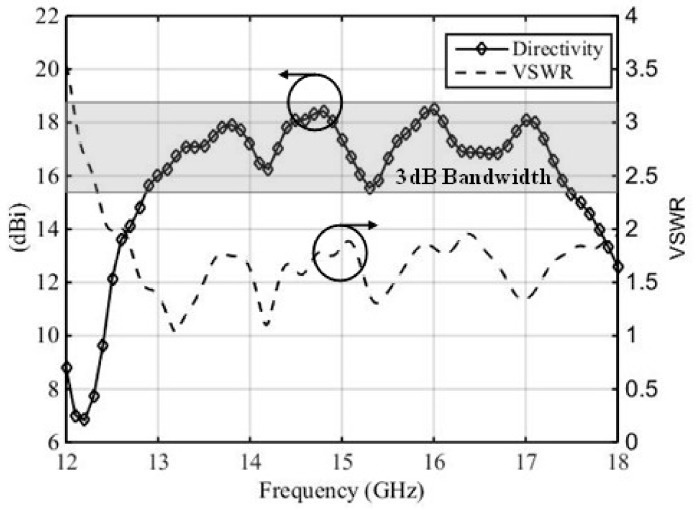
Broadside directivity and input matching of the RCA with planar stripline-fed U-slot.

Figure 4 shows the broadside directivity and VSWR of this RCA, with the planar feed interfaced with the wideband PRS. The peak directivity increased from 8 dBi (feed only, see Figure 2b) to 18.5 dBi in Figure 4, which is a significant 10 dB increase. The radiation pattern of the RCA with U-slot feeding structure at 15 GHz is also given in Figure 2e to show the improvement. The VSWR 2:1 bandwidth spans over 12.4–18 GHz, corresponding to 37% matched bandwidth. The matched bandwidth clearly complements the 3 dB directivity bandwidth, which is imperative to obtain an equivalent 3 dB gain bandwidth and to minimize the mismatch loss. It can be noted in Figure 4 that the curve of broadside directivity versus frequency is not entirely flat, and has some drops at 14.2 GHz, 15.3 GHz, and 16.5 GHz, although these drops are less than 3 dB from the peak value of directivity. A close comparison of Figure 4 with Figure 2b shows that these frequencies approximately correspond to the frequencies where the broadside directivity of the feed antenna drops down, which in turn reflects in the broadside directivity of RCA. As discussed in Section 2, the air gap h_1_, and the dimensions of the U-shaped stub at the coupling end of the stripline, were parametrically studied to achieve the best compromise between matching and broadside directivity. These two parameters can readily influence the 3-dB directivity bandwidth, particularly when there a small drops observed in the broadside directivity, seen in Figure 4.

**Figure 5:**
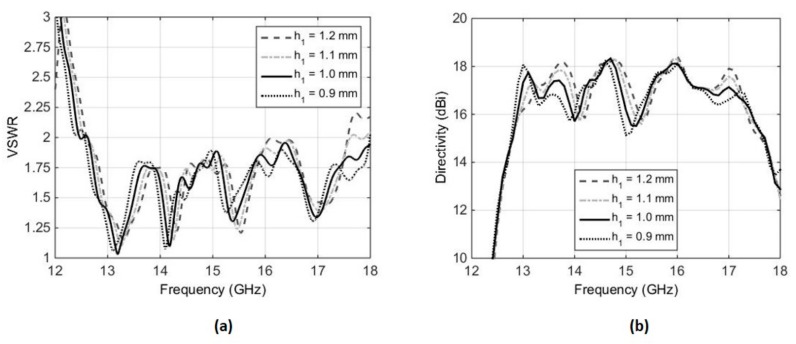
Sensitivity of air-gap h_1_ with respect to VSWR and broadside directivity of the RCA, (**a**) VSWR, (**b**) broadside directivity.

**Figure 6:**
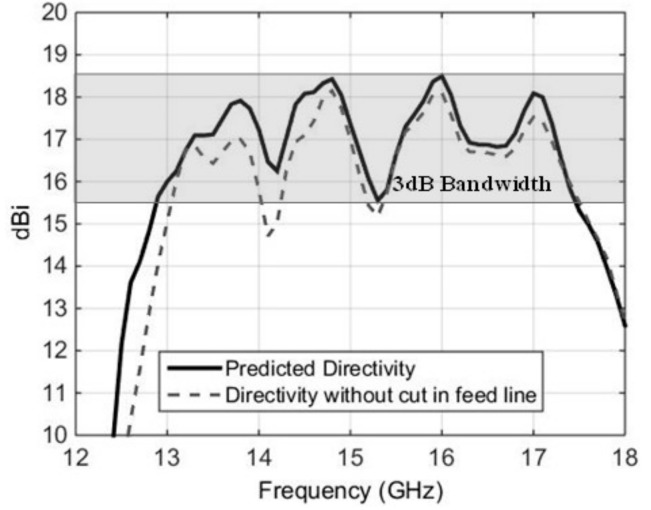
Effect of U-shaped stub in increasing the directivity bandwidth of the antenna.

Figure 5 shows the effect of slight variations in the air gap h_1_, by varying the value of h_1_ from 0.9 mm to 1.2 mm. It can be observed from Figure 5 that larger values of h_1_ may lead to a deterioration in the matching of the RCA (see Figure 5a), particularly from 16–16.5 GHz and 17.5–18 GHz, whereas smaller values of h_1_ can influence the drop in broadside directivity around 15 GHz, and has the potential of splitting the 3 dB directivity bandwidth into two bands (see Figure 5b). Therefore, setting the value of h_1_ = 1mm is a reasonable compromise. Similarly, Figure 6 shows the effect of a U-shaped stub that is added and tuned at the coupling end of the stripline. In the absence of this stub, the broadside directivity of the feed antenna is reduced and, in turn, the broadside directivity of the RCA is decreased. Figure 6 shows that, with the U-shaped stub removed, the directivity bandwidth reduces from 32% (12.8 to 17.65 GHz) to only 21% (14.2 to 17.6 GHz).

## 4. Measurement

**Figure 7:**
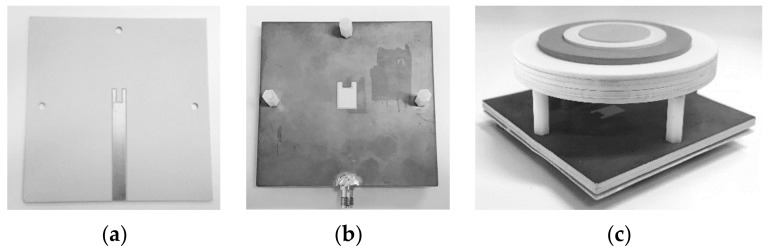
Photograph of fabricated prototype for measurements. (**a**) Back view, (**b**) top view of substrates of planar wideband feed taken apart and (**c**) the assembled RCA prototype.

A prototype of the proposed RCA interfaced with the planar feed was fabricated and measured in the NSI-700S-50 spherical nearfield anechoic chamber. Three nylon spacers were used to mount the wideband dielectric PRS above the planar feed and a SMA connector was soldered to the stripline. The prototype is shown in Figure 7, along with the stripline assembly taken apart to show the U-shaped stub, and the U-slot etched in the ground plane. The U-slot was etched on one side of the copper cladding on the Rogers RO3003 substrate (Rogers Corporation Inc., Chandler, AZ, USA) (ε_r_ = 3.0 and tan δ = 0.0019) having a thickness of 3.04 mm. The stripline was laid out on the bottom of the substrate, and excess cladding was etched out, as shown in Figure 7. The U-slot feeding structure and dielectric PRS were constructed using the parameters given in Table 1 and Table 2, respectively.

**Figure 8:**
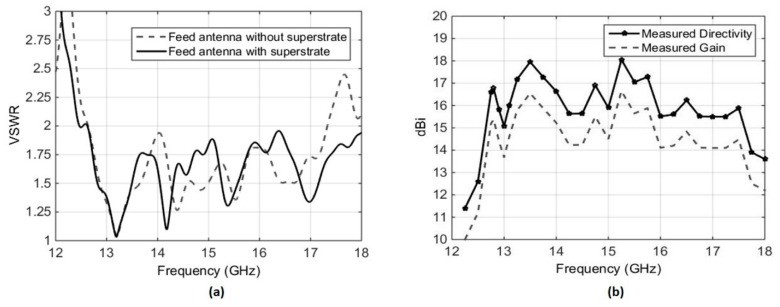
Measured results of the RCA with planar feed antenna, (**a**) VSWR of the antenna with and without PRS, (**b**) broadside directivity and realized gain.

The VSWR of the planar feed antenna was measured using an Agilent PNA-X N5242A Vector Network Analyzer (Agilent Technologies Inc.). Figure 8a shows the VSWR for the stand-alone planar feed, as well as with the planar feed interfaced with the dielectric PRS. It can be seen that, for both cases, the VSWR is less than 2 over the frequency range from 12.3 GHz to 17.8 GHz. In case of the standalone feed antenna, the VSWR rises above 2 beyond 17.25 GHz. This is because the U-slot and the U-shaped stub were optimized with the PRS integrated with the feed antenna.

**Figure 9:**
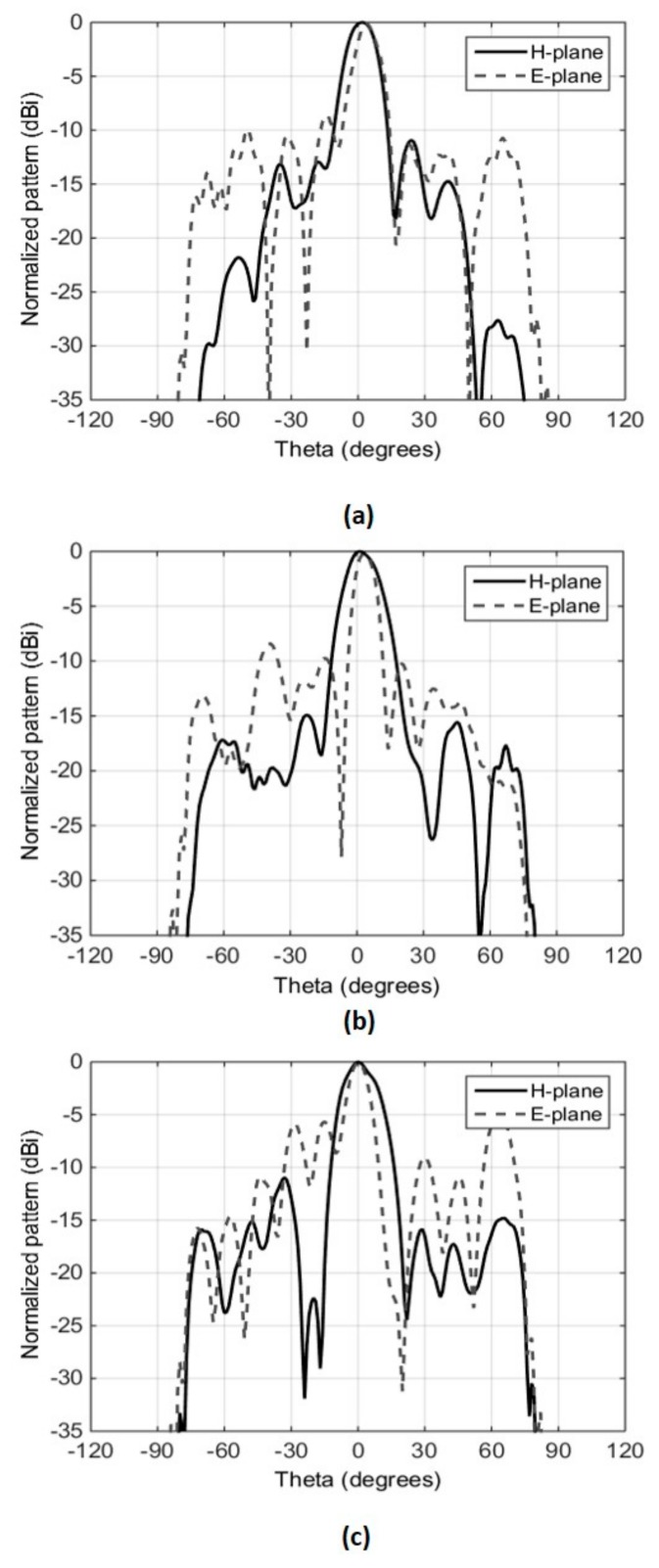
Radiation patterns of the RCA with planar feed measured at Australian Antenna Measurement Facility (AusAMF), (**a**) 12.8 GHz, (**b**) 15.0 GHz, (**c**) 16.0 GHz.

The prototype demonstrated a measured peak broadside directivity and realized gain of 18 dBi and 16.7 dBi, respectively, with a measured 3 dB directivity bandwidth extending from 12.3 GHz to 17.8 GHz, as shown in Figure 8b. The gain was measured using the gain comparison method, using standard-gain horns SGH-75 and SHG-51 as the reference antennas in the respective frequency bands. A slight difference between directivity and gain, approaching a maximum of 1.5 dB at a few data points, can be observed in Figure 8b. This difference is attributed to two facts. Firstly, the gain-comparison method presents an inherent tolerance of ±0.5 dB. Secondly, the VSWR of the antenna is very sensitive to the air gap in the planar feed antenna, as discussed in Section 3. While all due care was taken to maintain the air gap adequately and to keep the bottom and top layers of the feed parallel to each other, the prototype is not perfect. The measured radiation patterns of the presented antenna at three different frequencies within the gain bandwidth, shown in Figure 9, confirm the directive nature of the antenna.

The measured performance comparisons of the proposed antenna with the previously reported RCA is given in Table 3. As shown, the proposed antenna demonstrated significantly large bandwidth compared to that of the RCA fed by a slot-coupled WR-75 waveguide [4] and slot coupled patch antenna [10]. 

## 5. Conclusions

A simple, planar, wideband feed antenna has been proposed, which can be used to feed wideband RCAs. It has the potential to be fabricated through the standard printed-circuit board (PCB) manufacturing process, and can easily be interfaced with any type of wideband PRS to form a stand-along RCA. The approach is a simple alternative to the expensive and bulky waveguide feeding methods used in conventional wideband RCAs. A maximum antenna gain of 16.7 dBi has been obtained with a directivity bandwidth covering nearly the entire Ku-band. Measured results of the fabricated porotype are presented and demonstrate a wideband performance with reasonably high gain and excellent matching.

## Figures and Tables

**Table 1:** Parameter values for the stripline feed structure (in mm).

Parameter	*w_S_*	*w_SS_*	*w_L_*	*w_LL_*	*f_W_*
	9.0	4.5	13.125	2.875	6
Parameter	*f_SL_*	*f_SW_*	*sub_x_*	*sub_y_*	*f_L_*
	6.0	3.0	80.0	80.0	47.5

**Table 2:** Design parameters of the wideband dielectric partially reflecting superstructure (PRS) (in mm).

Parameter	*r_1_*	*r_2_*	*r_3_*	*r_4_*	*h*
	14	20	29.6	39.5	16
Parameter	*t_1_*	*t_2_*	*t_3_*	*t_4_*	*h_L_*
	11.9	11.4	10.16	8.18	1

**Table 3:** Measured performance comparison of the proposed antenna with previously published antennas.

	Feed Type	Peak Directivity (dBi)	Bandwidth (GHz/%)	Footprint (mm × mm)
This work	U-slot	18	12.3–17.8 GHz	80 × 80
[4]	WR-75	20	15%	80 × 80
[10]	Slot Coupled patch	15	13.5–17.5 GHz	45 × 45
